# Unusual Thermo‐Enhanced Second Harmonic Generation in Organic Configurationally‐Locked Polyene Crystals

**DOI:** 10.1002/advs.202412218

**Published:** 2024-11-24

**Authors:** Yi Liu, Pengfei Zhu, Qingshun Fan, Zihao Zhao, Linjie Wei, Yu Ma, Haojie Xu, Wuqian Guo, Junhua Luo, Zhihua Sun

**Affiliations:** ^1^ State Key Laboratory of Structure Chemistry Fujian Institute of Research on the Structure of Matter Chinese Academy of Sciences Fuzhou Fujian 350002 P. R. China; ^2^ University of Chinese Academy of Sciences Beijing Beijing 100049 P. R. China; ^3^ Fujian Science & Technology Innovation Laboratory for Optoelectronic Information of China Fuzhou Fujian 350108 P. R. China

**Keywords:** nonlinear optical crystal, organic configurationally‐locked polyene, phase transition, thermo‐enhanced second harmonic generation

## Abstract

To modulate nonlinear optical (NLO) effects of crystalline material holds great application potential in the photoelectronic and optical fields. Organic configurationally‐locked polyene represents an exciting NLO family with large second harmonic generation (SHG) effects, whereas it is a huge blank to switch and modulate their NLO property through external stimuli. For the first time, here present unusual thermo‐enhanced SHG activities are presented in a polyene‐based NLO compound, 2‐{3‐[2‐(4‐pyrrolidinphenyl)vinyl]‐5,5‐dimethylcyclohex‐2‐enylidene}malononitrile (**1**), giving a record‐high magnitude of SHG enhancement up to ≈170% during its isomorphic phase transition. Theoretical analysis discloses this behavior stems from the reduced degree of torsion in the *π*‐conjugated structures in **1**, as verified by dihedral angles between its pyrrolidine and phenyl planes. As the first study on thermo‐enhanced SHG properties of organic crystals, this work affords a new avenue of modulating physical properties to fabricate high‐performance photoelectronic and optical devices.

## Graphical abstract

The unprecedented thermo‐enhanced second harmonic generation (SHG) response is achieved in an organic configurationally‐locked polyene nonlinear optical (NLO) crystal, ascribed to the decrease of torsion degree of the *π*‐conjugated bridge induced by the molecular dynamic motions. This work paves a new pathway to develop smart photoelectronics devices.

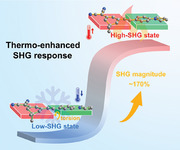

## 1 Introduction

Nonlinear optical (NLO) modulation of crystalline materials, showing an ability to commute reversibly between two or more different states, displays a contrast of their inherent NLO characteristics, typically the second‐harmonic generation (SHG) effect.^[^ ^1^ ^]^ Such modulation behaviors are usually triggered by external stimuli including light irradiation, temperature, pressure, or pH variation, which have great application potentials in photoelectronic and optical fields, such as data storage, signal processing, sensing, and electro‐optical switching.^[^ ^2^ ^]^ Emphatically, organic solid‐state NLO switches with outstanding and stable modulation of SHG responses can be achieved by adjusting the conformation or orientation of NLO chromophore molecules.^[^ ^3^ ^]^ Among them, thermal‐induced structural phase transition has been proven to be a feasible strategy verified by a variety of molecular‐based crystalline materials, such as organic‐inorganic hybrids, metal‐organic frameworks, host‐guest compounds, and ionic salts.^[^ ^4^ ^]^ The basic principle for these materials is the change of structural symmetry caused by the variable temperature, commonly transforming from a low‐temperature non‐centrosymmetric structure (i.e., SHG‐active) to a high‐temperature centrosymmetric structure (i.e., SHG‐inactive).^[^ ^5^ ^]^ In exceptionally rare cases, a unique phase transition in inorganic NLO oxide RbNaMgP_2_O_7_ enhances the SHG effect by heating.^[^ ^6^ ^]^ Contrarily, the enhancement and modulation of bulk NLO properties in complex organic systems remains quite challenging, due to the lack of knowledge on controlling the conformation or orientation of NLO‐active chromophore molecules through external stimuli.

Organic NLO materials have been widely used in diverse optical and photoelectronic devices due to their unique properties of high nonlinearities and low dielectric constants.^[^ ^7^ ^]^ These merits closely involve with the structure origin of the NLO responses, namely, the transfer of electronic charge across the molecules, which is different from inorganic counterparts that rely on geometric perturbations of the ions within the crystalline framework.^[^ ^8^ ^]^ In particular, long *π*‐conjugated polyene molecules are important members of the organic NLO family, and they usually have large molecular second‐order optical nonlinearity.^[^ ^9^ ^]^ In terms of structure, such *π*‐conjugated polyene molecules exhibit unique design possibilities; the electron donor and acceptor for a given conjugated bridge can be tailored to balance the electronic asymmetry and polarizability by virtue of coulomb interaction, hydrogen bonds, molecular asymmetry, and chirality, and further modulate second‐order NLO effects.^[^ ^10^ ^]^ The most well‐known example of organic NLO crystal is 4′‐dimethylamino‐*N*‐methyl‐4‐stilbazolium tosylate (DAST), which has a large macroscopic SHG effect but suffers from insufficient thermal stability.^[^ ^11^ ^]^ Another reported configurationally‐clocked polyene (CLP), 2‐(3‐(4‐hydroxystyryl)‐5,5‐dimethylcyclohex‐2‐enylidene)malononitrile (OH1), displays superior thermal stability and comparable second‐order nonlinearity to DAST.^[^ ^12^ ^]^ Remarkably, the dynamic organic component of the electron donor allows a large freedom of molecular motion, which offers the driving force for thermo‐induced structural phase transitions. Taking into consideration of mutual exclusion between high temperature and molecular asymmetry, however, it remains a challenge to control the molecular dynamics in organic crystals to achieve thermo‐induced SHG enhancement, along with a detailed understanding of this rare phenomena.

Herein, thermo‐induced SHG enhancement is explored in an organic CLP‐based NLO crystal, 2‐{3‐[2‐(4‐pyrrolidinphenyl)vinyl]‐5,5‐dimethylcyclohex‐2‐enylidene}malononitrile (**1**), which undergoes a reversible polar‐polar isomorphic phase transition at 358 K. The dynamic motions of pyrrolidine and phenyl groups reduce the torsion degree of its *π*‐conjugated bridge and thus achieve a record‐high magnitude of SHG enhancement up to ≈170% among the known solid‐state NLO materials (e.g., RbNaMgP_2_O_7_ ≈150%). Consequently, **1** is expected to become a strong candidate for new‐generation NLO crystalline material. The findings expand the family of NLO materials with enhanced SHG response and provide new insights toward the design of future smart photoelectronic and optical device applications.

## 2 Results and Discussion

**Figure 1:**
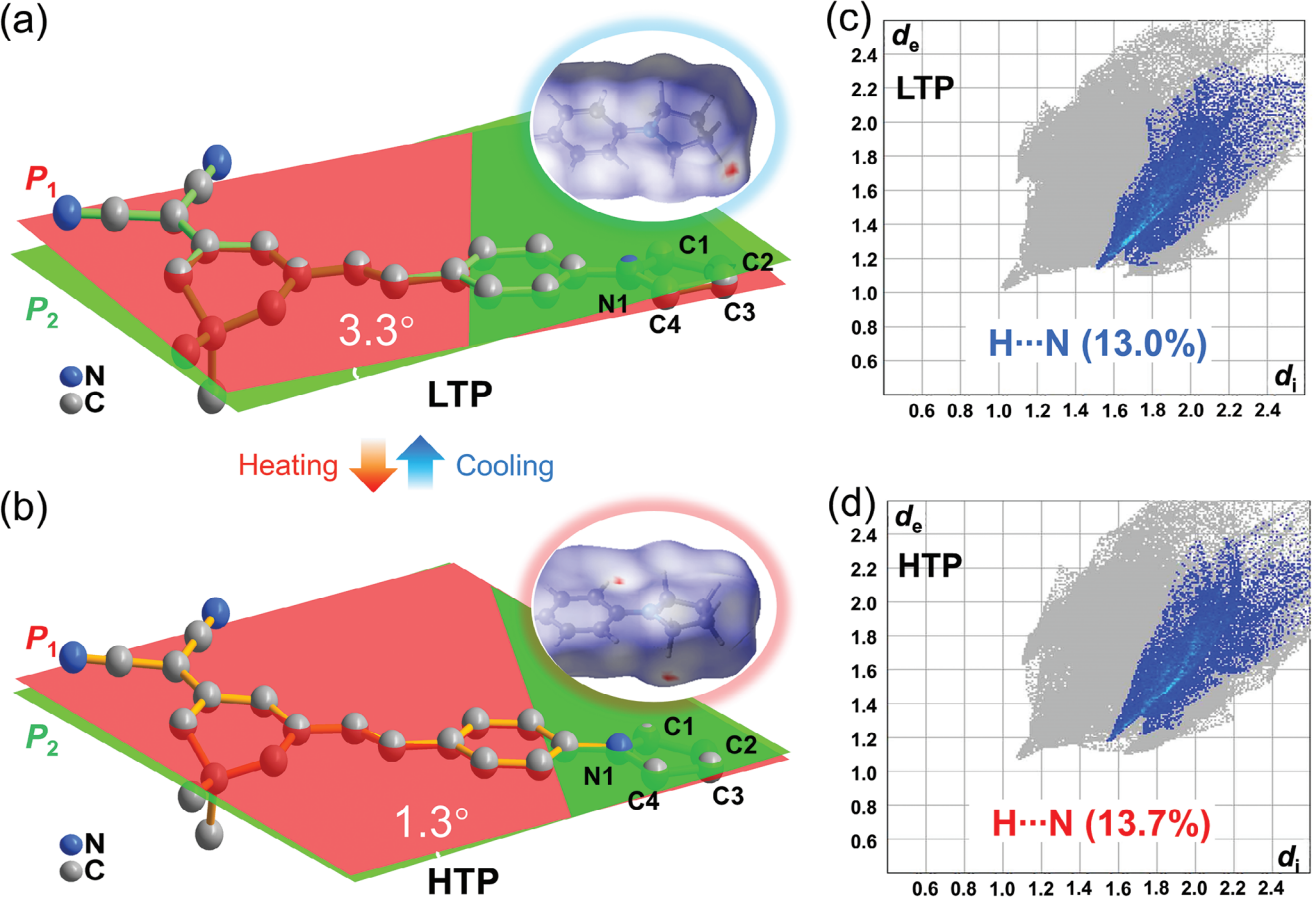
Molecular structures of **1** at a) LTP and b) HTP, respectively. The circles depicted the Hirshfeld surfaces mapped over *d*_norm_. The 2D fingerprint plots for **1** at c) LTP and d) HTP.

**1** was synthesized by the Knoevenagel condensation reaction of isophorone, malononitrile, and 4‐(1‐pyrrolidino)benzaldehyde (Figure S1, Supporting Information).^[^ ^13^ ^]^ Block single crystals of **1** were successfully prepared from methanol by the slow evaporation method (Figure S2, Supporting Information), and the purity was further verified by powder X‐ray diffraction measurements (Figure S3, Supporting Information). Crystal structures were resolved at 298 K (LTP, low‐temperature phase) and 373 K (HTP, high‐temperature phase) to confirm the origin of the phase transition. In LTP, **1** crystallizes in the polar space group *P*2_1_, as previously reported in the literature.^[^ ^14^ ^]^ As descripted in **Figure** **1a**, the phenyl ring corresponding to the plane *P*_1_ is slightly out of plane *P*_2_ from the main *π*‐conjugated bridge with a twist angle of 3.3°. Such nearly planar *π*‐conjugated bridge can promote an efficient *π*‐electron delocalization and thus achieve a strong SHG response. In addition, the pyrrolidine ring adopts non‐planar conformation with a torsion angle of 7.5°; planes *P*_C1‐N1‐C4_ and *P*_C3‐C4‐N1_ represent planes passing through C1‐N1‐C4 and C3‐C4‐N1 bonds, respectively (Figure S4a, Supporting Information). The remarkable intermolecular interactions, represented by the red circular depressions on the *d*_norm_ surfaces, can be derived by the 3D Hirshfeld surface analysis.^[^ ^15^ ^]^ As shown in Figure 1c, the 2D fingerprint plot of **1** shows that C‐H···N hydrogen‐bonding interaction between adjacent **1** molecules distributed over with 13.0% of the surface area. Figure 1b and Table S1 (Supporting Information) show that the HTP is isostructural to the LTP one. The transition between the two phases is accompanied by changes in neither the crystallographic space group nor the disorder, but the lattice parameters change obviously. Subsequently, the temperature dependence of local configuration and molecular interactions of **1** was investigated. From LTP to HTP, the torsion angle of pyrrolidine rings increases to 12.1° (Figure S4b, Supporting Information); the twist angle between the phenyl ring and the main *π*‐conjugated bridge transforms to 1.3°. From Hirshfeld surfaces, the positions of significant molecular interaction contacts have changed obviously with the increasing temperature, and are mainly concentrated on the pyrrolidine and phenyl rings (Figure 1b,d). Obviously, the origin of reversible phase transitions is probably attributed to the dynamic motion of pyrrolidine and phenyl groups, which provides an opportunity for thermo‐induced SHG enhancement.

**Figure 2:**
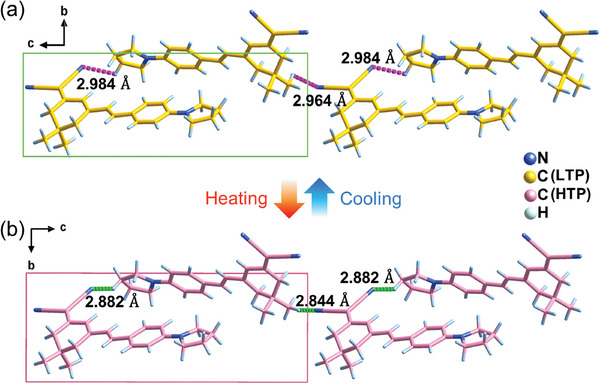
Crystal packing diagram projected along the *a*‐axis. The C─H···N hydrogen‐bonding interactions between adjacent **1** molecules at a) LTP and b) HTP, respectively.

Figure S5 (Supporting Information) shows the unit cell of **1** projected along the *b*‐axis of the crystal. The long axis of the **1** is aligned at an angle of 30° relative to the *c*‐axis in LTP, and this angle changes to 45° in HTP. For that reason, we performed the temperature dependences of the refined cell parameters between 298 and 373 K. Clearly, as the temperature increases to *T*_c_, the crystal expands along the *a*‐ and *b*‐axes with the linear compressibility of ‐26% and ‐1.2%, respectively. In contrast, the *c*‐axis and *β* angle are decreased from 21.73 to 20.38 Å (by 6.2%) and from 97.45 to 92.63° (by 4.9%), respectively (Figure S6, Supporting Information). It is worth noting that all the **1** molecules have similar arrangements with the pyrrolidine and cyanide groups aligning along the *b*‐axis directions, resulting in its electric polarization. There are strong intermolecular hydrogen bonds between the neighboring **1** molecules, in which the N atoms on the cyanide groups act as hydrogen‐bond acceptors with H‐bond parameters of ∠C‐H···N ≈115.0–120.7° and *d*C‐H···N ≈2.964–2.984 Å. From LTP to HTP, the *d*C‐H···N reduced to ≈2.844–2.882 Å and was accompanied by the change of orientation (**Figure** **2**a,b). The highest macroscopic second‐order nonlinear optical response *d*_333_ can be roughly calculated by formula *d*_333_ = *N* β *f*(ω) <cos^3^ *θ*_p_>, where *N* and β are the number of molecules per unit volume and the microscopic molecular hyperpolarizability, respectively; *f*(ω) is the local field factor, and *θ*_p_ is the angle between the molecular charge‐transfer axis and the polar crystalline axis.^[^ ^16^ ^]^ Hence, the SHG efficiency of **1** crystal can be related to the order parameter of the polar structure cos *θ*_p_. Figure S7 (Supporting Information) shows that the long axis of **1** is a favorable direction of charge delocalization. Obviously, HTP is more aligned along the crystallographic polar *b*‐axis than that of LTP, which leads to the observed enhancement in the macroscopic NLO effect.

**Figure 3:**
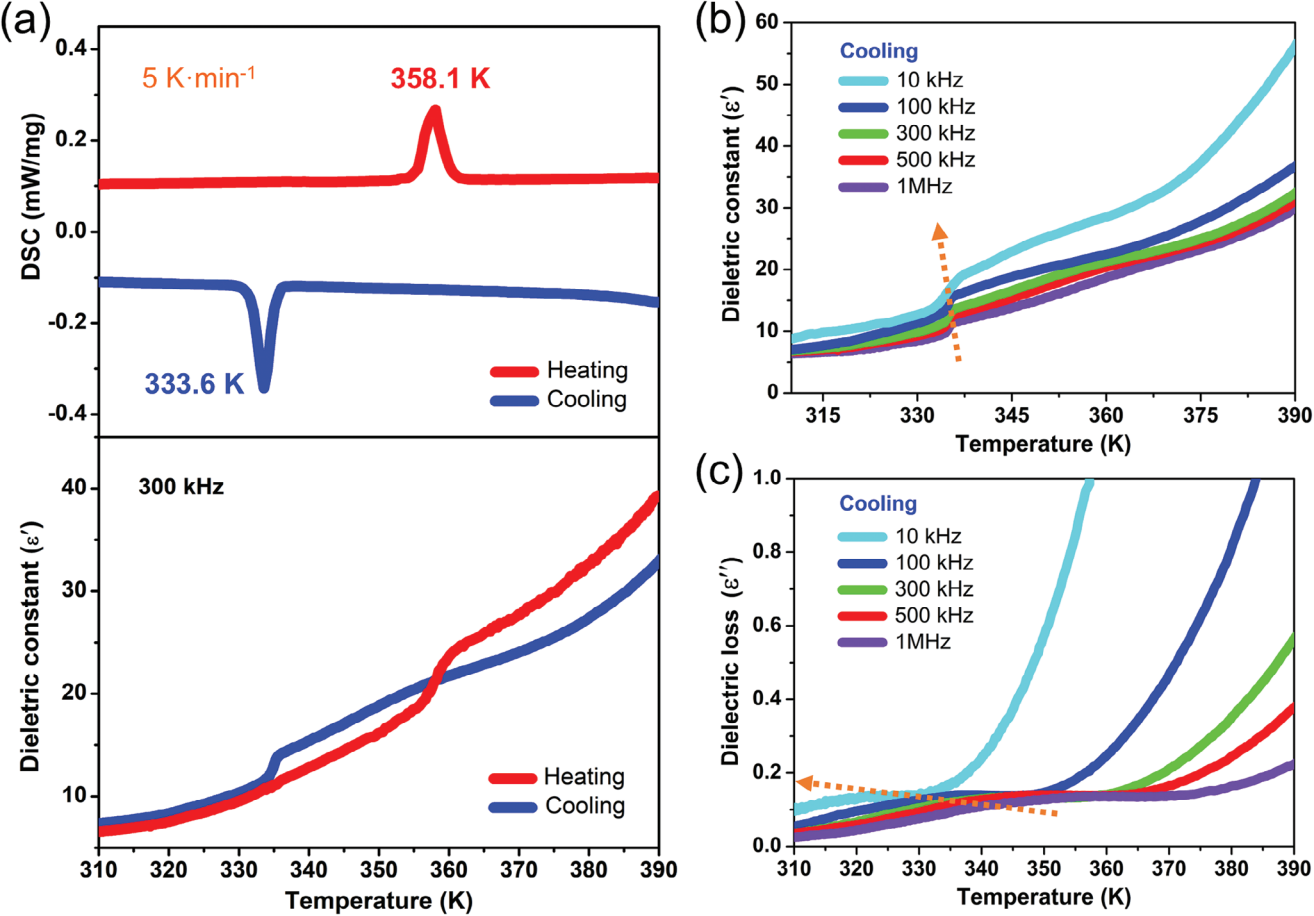
Phase transition behaviors of **1**. a) DSC curves measured in a heating‐cooling cycle at a rate of 5 K·min^−1^ (top). The temperature dependence of the *ε*ʹ measured in a heating‐cooling cycle (bottom). Frequency dependence of (b) the *ε*ʹ and (c) *ε*′′ in the cooling cycle, respectively.

Differential scanning calorimetry (DSC) measurements reveal a pair of reversible thermal anomaly peaks centered ≈358.1 333.6 K^−1^ (*T*_c_) in the heating/cooling runs with the large heat hysteresis of ≈24.5 K, further verifying the first‐order phase transition of **1**, as shown in **Figures** **3**a and S8 (Supporting Information). Estimated transition enthalpy (Δ*H*) and entropy change (Δ*S*) are ≈0.5 kJ·mol^−1^ and 1.4 J·mol^−1^·K^−1^, respectively. According to the Boltzmann equation, Δ*S* = *R* ln *N*, the number of molecular orientations *N* is approximately calculated as 1.2, which is much less than 2.0, indicating the first‐order phase transition characteristics of **1**.^[^ ^17^ ^]^ The thermogravimetric measurement indicates that **1** shows a high thermal stability up to ≈450 K, ensuring its high‐temperature phase transition (Figure S9, Supporting Information). In general, the dielectric anomaly is probably suggestive of crystal structure transformation. Here, the temperature‐dependent dielectric constant (*ε*′) and loss (*ε*′′) were measured in the temperature ranges of 310–390 K at the different frequencies. The steep dielectric anomaly platforms emerge at *T*_c_, coinciding well with the result determined by DSC measurement. As shown in Figure 3b,c, the dielectric anomalies exhibit the characteristic feature of frequency dispersion,^[^ ^18^ ^]^ in which the anomaly platforms in *ε*′ and *ε*′′ shift toward higher temperature as frequency increases from 10 kHz to 1 MHz.

**Figure 4:**
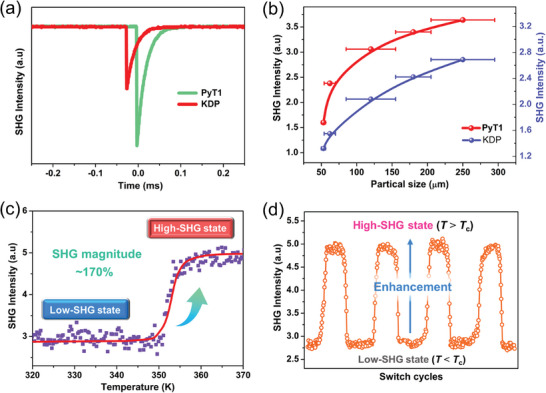
a) Experimental SHG signals for **1** and KDP collected at room temperature. b) The phase matching curves of **1** as compared to the KDP reference. c) Temperature‐dependent SHG signals upon heating. d) Recoverable SHG enhancement between high‐ and low‐SHG states.

Prior to the investigation of temperature‐dependent SHG signals, the SHG intensities of **1** were measured under the solid‐state Q‐switched OPO laser irradiation of 2100 nm at room temperature. The SHG intensity of LTP is ≈1.2 times that of the benchmark KH_2_PO_4_ crystal with the same particle size (**Figure** **4**a). Besides, **1** shows the phase‐matchable property, that is, the enhancement of SHG intensities with the increasing of particle sizes. (Figure 4b).^[^ ^19^ ^]^ As shown in Figure 4c, the SHG signals maintain nearly constant until *T*_c_ ≈ 358 K, corresponding to the low‐SHG state. These figure‐of‐merits are higher than those of some reported solid‐state NLO switch materials, such as [Hdabco]^+^[CF_3_COO]^−^ (SHG intensity ≈ 1 × KH_2_PO_4_, *T*_c_ ≈ 180 K),^[^ ^3b^ ^]^ (Me_3_NNH_2_)_2_[CdI_4_] (SHG intensity ≈0.5 × KH_2_PO_4_, *T*_c_ ≈213 K)^[^ ^4c^ ^]^ and [C_4_H_10_N][CdCl_3_] (SHG intensity ≈0.4 × KH_2_PO_4_, *T*_c_ ≈240 K).^[^ ^15a^ ^]^ As the temperature continues to rise, the SHG signals jump evidently around *T*_c_ and then completely stay at the high‐SHG state. For phase transition compounds, their symmetry of structure generally increases with the raising temperature; the corresponding NLO‐switching behavior is to quench SHG by heating. In this work, **1** undergoes an isomorphic polar‐polar isomorphic phase transition, accompanied by a very rare SHG enhancement with an evident SHG magnitude up to ≈170%. This SHG contrast is higher than that of rarely reported inorganic NLO oxide RbNaMgP_2_O_7_ (≈150%).^[^ ^6^ ^]^ Moreover, the NLO responses show a rapid and quite repeatable features without any attenuation after multiple cycles, indicating its potential in NLO tunable device application (Figure 4d).

**Figure 5:**
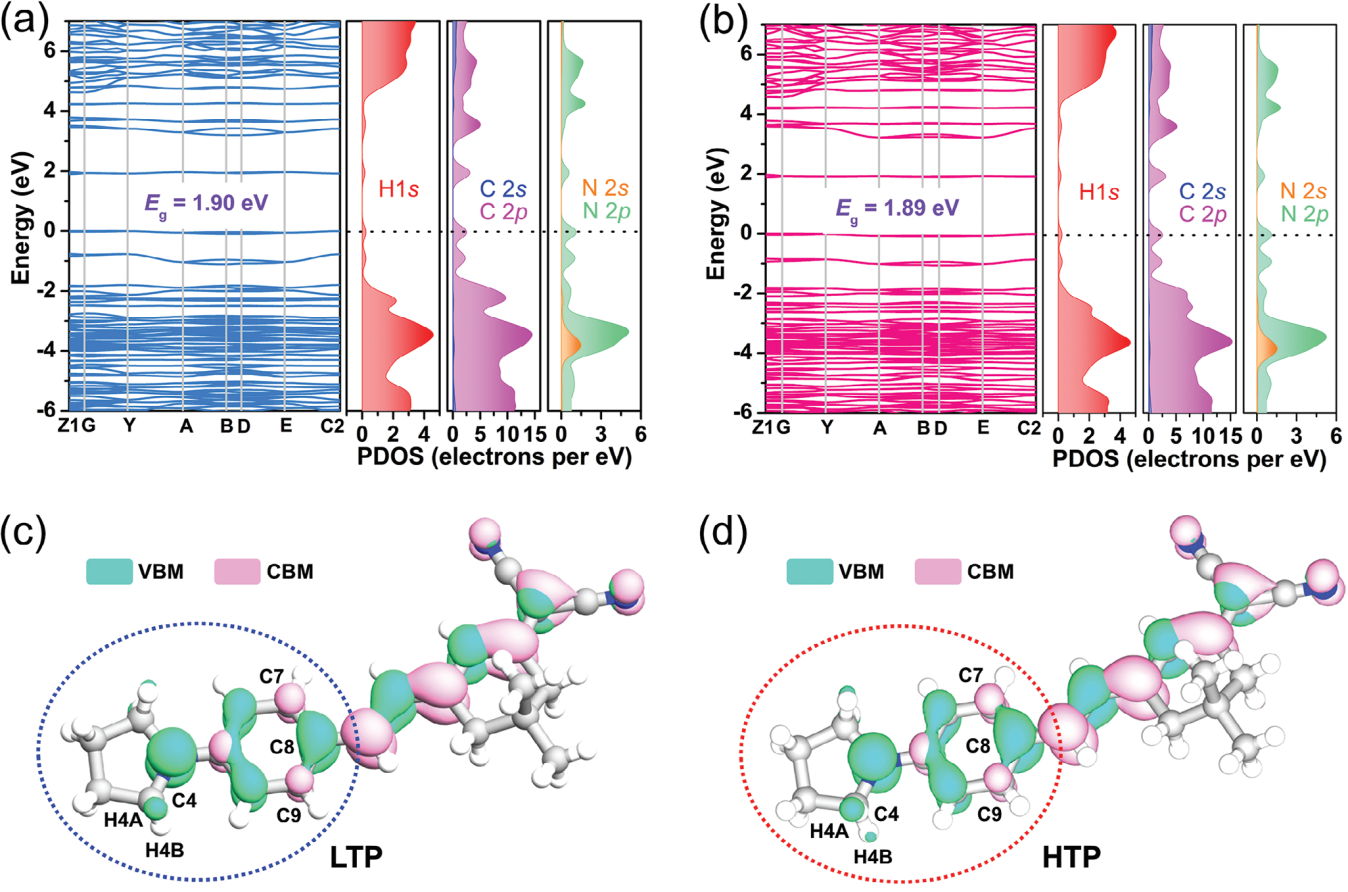
Electronic band structure and partial density of states of **1** in a) LTP and b) HTP. Charge density isosurfaces for HOMO (green) and LUMO (pink) orbitals of **1** in c) LTP and (d) HTP.

In order to further confirm the structure origin of the thermo‐induced SHG enhancement of **1**, its electronic structures in the LTP and HTP were analyzed. Density functional theory (DFT) calculations indicate that both the LTP and HTP of **1** have a narrow band gap of ≈1.90 eV (**Figure** **5**,b), which coincides with the sharp absorption edge at a wavelength of 700 nm (Figure S10, Supporting Information).^[^ ^20^ ^]^ As shown in the partial density of states (PDOS) spectra for **1**, the H‐1*s*, N‐2*p*, and C‐2*p* orbitals determine the conduction‐band minimum (CBM) and valence‐band maximum (VBM). This result demonstrates notable hybridization between N and C ions, representing the delocalized *π*‐conjugation of the benzene ring and C═C groups. From its lowest unoccupied molecular orbitals (LUMO) and highest occupied molecular orbitals (HOMO), the electron density of **1** is mainly distributed on the benzene ring and C═C groups (Figure 5c,d). It is obvious that the electron density in the HTP displays distinct variations with them in the LTP. Particularly, the overlapped electron cloud density at both sides of the C7‐C8‐C9 bond is increased from LTP to HTP, suggesting an enhancement of 𝜋‐delocation; the electron clouds of H4A and H4B atoms are also changed. These results are consistent with the dynamic motion of pyrrolidine and phenyl groups across the phase transition. In contrast, the variations in the C─H···N distance and molecular orientation are slight. Besides, the phenyl rings exhibit strong structural rigidity. Tables S2 and S3 (Supporting Information) show that the twist angle of phenyl rings remains constant as the temperature increases. Therefore, such thermo‐induced SHG enhancement can be primarily attributed to the transformation of torsion degree in the *π*‐conjugated structures.

## 3 Conclusion

In summary, we report the thermo‐induced SHG enhancement in an organic CLP, 2‐{3‐[2‐(4‐Pyrrolidinphenyl)vinyl]‐5,5‐dimethylcyclohex‐2‐enylidene}malononitrile (**1**), which exhibits a reversible polar‐polar phase transition at 358 K. Particularly, the dynamic motion of pyrrolidine and phenyl groups behaves as the driving force to reduce the degree of torsion of its *π*‐conjugated bridge, thus achieving the enhancement of SHG response. Such unusual NLO modulation behavior endows a high magnitude of SHG enhancement up to ≈170%. To the best of our knowledge, this is the first organic polyene‐based NLO compound that shows such a fascinating thermo‐enhanced SHG response induced by the polar‐polar isomorphic phase transition. The findings will be advantageous to the progress of NLO modulation applications and shed light on the rational design of new organic NLO candidates.

## Conflict of Interest

The authors declare no conflict of interest.

## Supporting information



Supporting Information

## Data Availability

Research data are not shared.
