# Supplementary material for: Unusual Thermo‐Enhanced Second Harmonic Generation in Organic Configurationally‐Locked Polyene Crystals
Source: Adv Sci (Weinh). 2024 Nov 24;12(3):2412218. doi: 10.1002/advs.202412218 (PMC11744723; doi:10.1002/advs.202412218)
Supplement: Supplementary file 1 — Supporting Information [file ADVS-12-2412218-s001.docx]

**Supporting Information**

**Unusual Thermo-Enhanced Second Harmonic Generation in Organic Configurationally-Locked Polyene Crystals**

*Yi Liu, Pengfei Zhu, Qingshun Fan, Zihao Zhao, Linjie Wei, Yu Ma, Haojie Xu, Wuqian Guo, Junhua Luo and Zhihua Sun,**

Experimental Section

Synthesis and single-crystal growth. The target compound 1 was prepared by mixing stoichiometric ratio of isophorone (1.382 g, 10 mmol), malonodinitrile (660 mg, 10 mmol) and 4-(1-pyrrolidino)benzaldehyde (1.75 g, 10 mmol) in the solution of methanol. Piperidine was used as a catalyst. After the continuous stirring for two days at 353 K, a clear red solution was obtained. At the beginning of the crystal growth, the saturated methanol solution of 1 was prepared at 315 K and then kept eight hours at 320 K. Needle-like red crystals of 1 were obtained by the temperature cooling method from the saturated solution with a cooling rate of 0.2 K/day, as shown in Figure S2.

Single-Crystal X-ray Crystallography and Powder X-ray Diffraction. We used the XtaLAB Synergy R, HyPix diffractometer with the Cu-*Kα* radiation (*λ* = 1.54184 Å) to collect X-ray diffraction data. The structures were solved by direct methods and confirmed by the full-matrix least-squares refinements on *F*^2^ using the *SHELXTL* software packing. All non-H atoms were refined anisotropically, and all H atoms were generated by the geometrical method and refined by using a “riding” model with *Uiso* = 1.2 *Ueq* (C). The above-mentioned structure solution and refinement were conducted in the *Olex*2 software. Crystal data for 1 at 298 and 373 K are listed in Table 1. Powder X-ray diffractometry (PXRD) data was recorded on the MiniFlex 600 X-ray diffractometer equipped with a Cu *K*α radiation.

**Characterization.** The dielectric analyses were performed on TongHui TH2828 analyzer in the temperature range of 310-390 K. Single-crystal plates of **1** with the surface deposited by silver conduction paste were used for dielectric measurements. DSC measurement of **1** was recorded by using a NETZSCH DSC 200F3 instrument in the temperature range of 310-390 K. The powder samples that placed in aluminum crucibles were heated and cooled with the rate of 2-10 K∙min^-1^ under a nitrogen atmosphere. Thermogravimetric measurement was carried out by STA449C Thermal Analyser in the temperature range of 300-1200 K. Analyzing of NLO was performed on crystal samples with Edinburgh Instruments of FLS 920, using the unexpanded laser beam (Vibrant 355 II, OPOTEK) with low divergence (pulsed OPO laser at 2100 nm). The potassium phosphate monobasic (KDP) with a particle size of ≈ 120 µm was used for comparison. The temperature during the measurements was controlled by a Linkam TS1500 at a rate of 10 K∙min^-1^. The UV absorption spectrum in the solid state was measured at room temperature on a PE Lambda 900 UV-visible-NIR spectrophotometer. Band structures and partial density of states were calculated with single crystal structure data of by Cambridge Sequential Total Energy Package (CASTEP) in the framework of DFT. The exchange-correlation potential was calculated using the Perdew-Burke-Ernzerhof for solids (PBEsol) function within the generalized gradient approximation (GGA). The interactions between the ionic cores and the electrons were characterized by the norm-conserving pseudopotential.

**Entropy change, enthalpy change and *N* value calculated from the DSC curve.**

***T* = 358.1 K**：

Δ*S* =$\int_{\text{T}_{\text{1}}}^{\text{T}_{\text{2}}} \frac{\text{Q}}{\text{T}}$ $T$ *dT*

≈ $\frac{\text{∆}\text{H}}{\text{T}_{\text{c}}}$

=$\frac{\text{1.4}\text{74}\text{ J }\text{g}^{\text{-1}}\text{ }\text{×}\text{ }\text{343.46}\text{ g}\text{ mol}^{\text{-1}}}{\text{3}\text{58.1}\text{ K}}$

= 1.414 J${\text{ }\text{mol}}^{\text{-1}}\text{ }\text{K}^{\text{-1}}$

Δ*S* = *R* ln *N*

*N =* exp{$\frac{\text{∆}\text{S}}{\text{R}}$} = exp{$\frac{\text{1.414} \text{J }\text{mol}^{\text{-1}}\text{ }\text{K}^{\text{-1}}}{\text{8.314} \text{J }\text{mol}^{\text{-1}}\text{ }\text{K}^{\text{-1}}}$}

= 1.185

Δ*H* = *T*Δ*S*

= 358.1 K × 1.414 J${\text{ }\text{mol}}^{\text{-1}}\text{ }\text{K}^{\text{-1}}$

= 506.4 J$\text{ mol}^{\text{-1}}$

***T* =333.6 K**：

Δ*S* =$\int_{\text{T}_{\text{1}}}^{\text{T}_{\text{2}}} \frac{\text{Q}}{\text{T}}$ *dT*

≈ $\frac{\text{∆}\text{H}}{\text{T}_{\text{c}}}$

=$\frac{\text{1.4}\text{94}\text{ J }\text{g}^{\text{-1}}\text{ }\text{×}\text{ }\text{343.46}\text{ g}\text{ mol}^{\text{-1}}}{\text{3}\text{33.6}\text{ K}}$

= 1.538 J$\text{mol}^{\text{-1}}\text{ K}^{\text{-1}}$

Δ*S* = *R* ln *N*

*N =* exp{$\frac{\text{∆}\text{S}}{\text{R}}$} = exp{$\frac{1.538 \text{J }\text{mol}^{\text{-1}}\text{ }\text{K}^{\text{-1}}}{8.314 \text{J}\text{ mol}^{\text{-1}}\text{ K}^{\text{-1}}}$}

= 1.203

Δ*H* = *T*Δ*S*

= 333.6 K × 1.538 J${\text{ }\text{mol}}^{\text{-1}}\text{ }\text{K}^{\text{-1}}$

= 513.1 J$\text{ mol}^{\text{-1}}$

[CCDC 2385524 and 2378226 contain the supplementary crystallographic data for this paper. These data can be obtained free of charge from The Cambridge Crystallographic Data Centre via www.ccdc.cam.ac.uk/data_request/cif.]

**Figure**


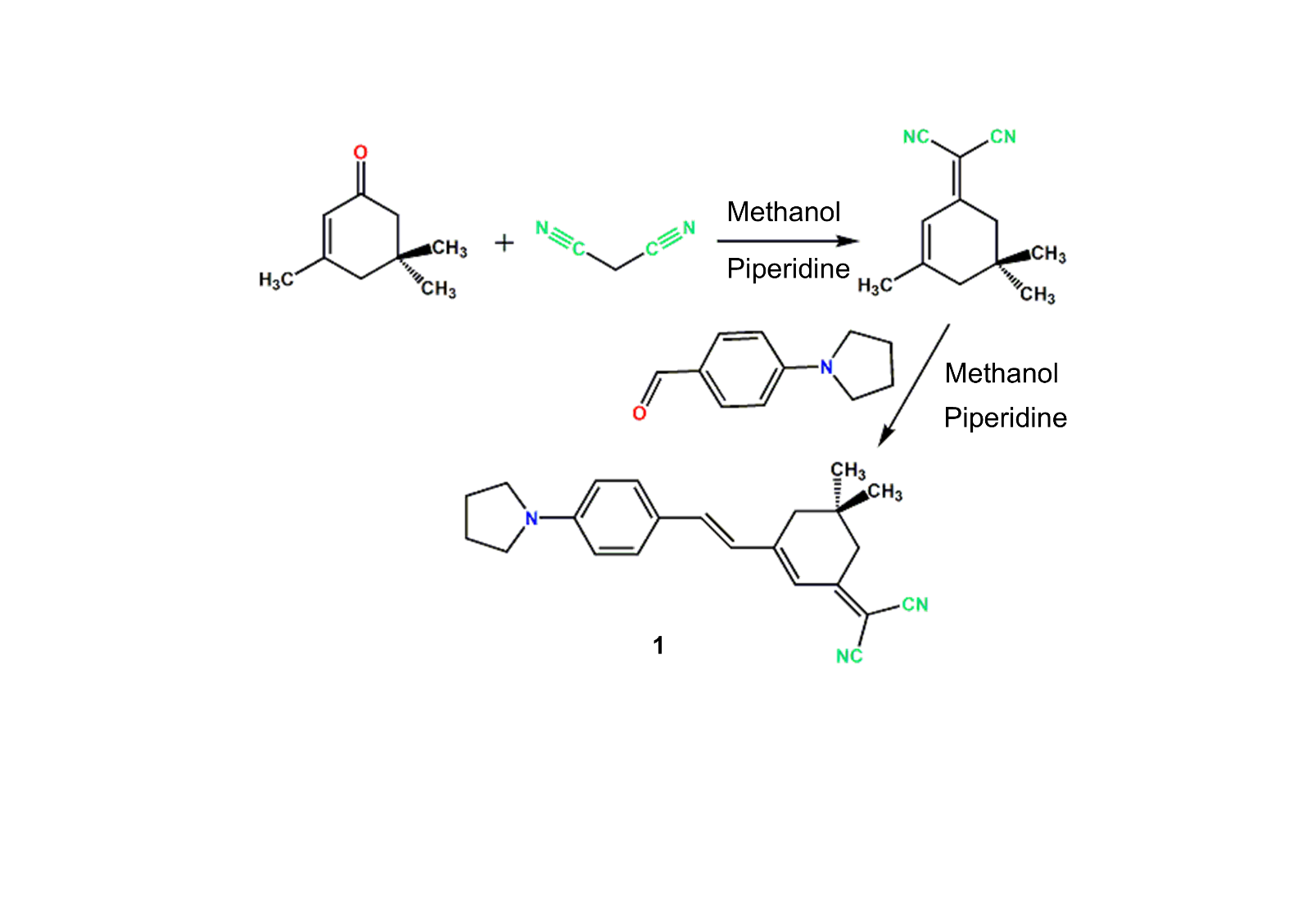


**Figure S1.** Synthetic scheme for **1**.


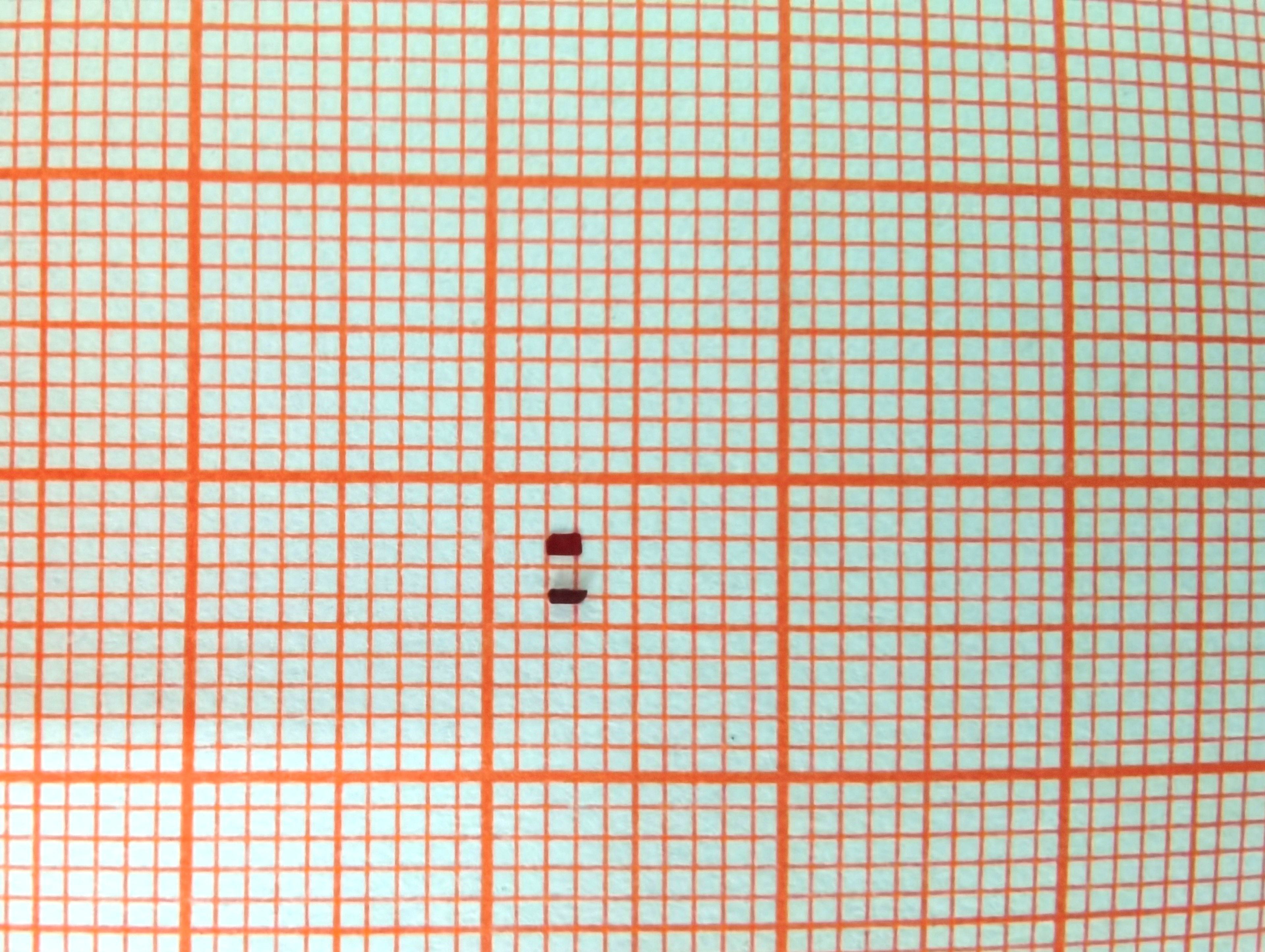


**Figure S2.** Needle-like single crystal of **1** obtained by the temperature-cooling method.


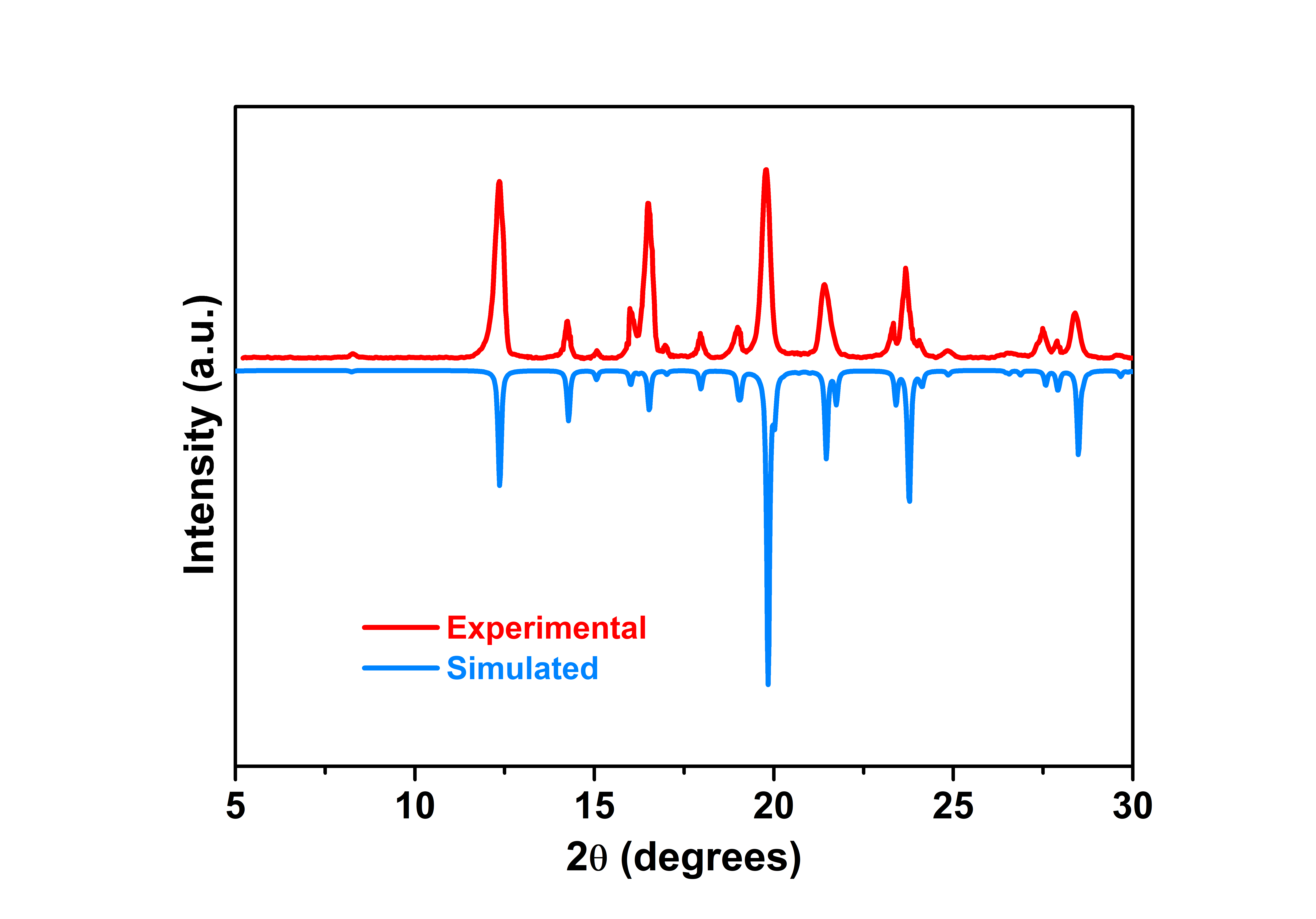


**Figure S3.** Experimental and simulated PXRD patterns for **1** obtained at room temperature.


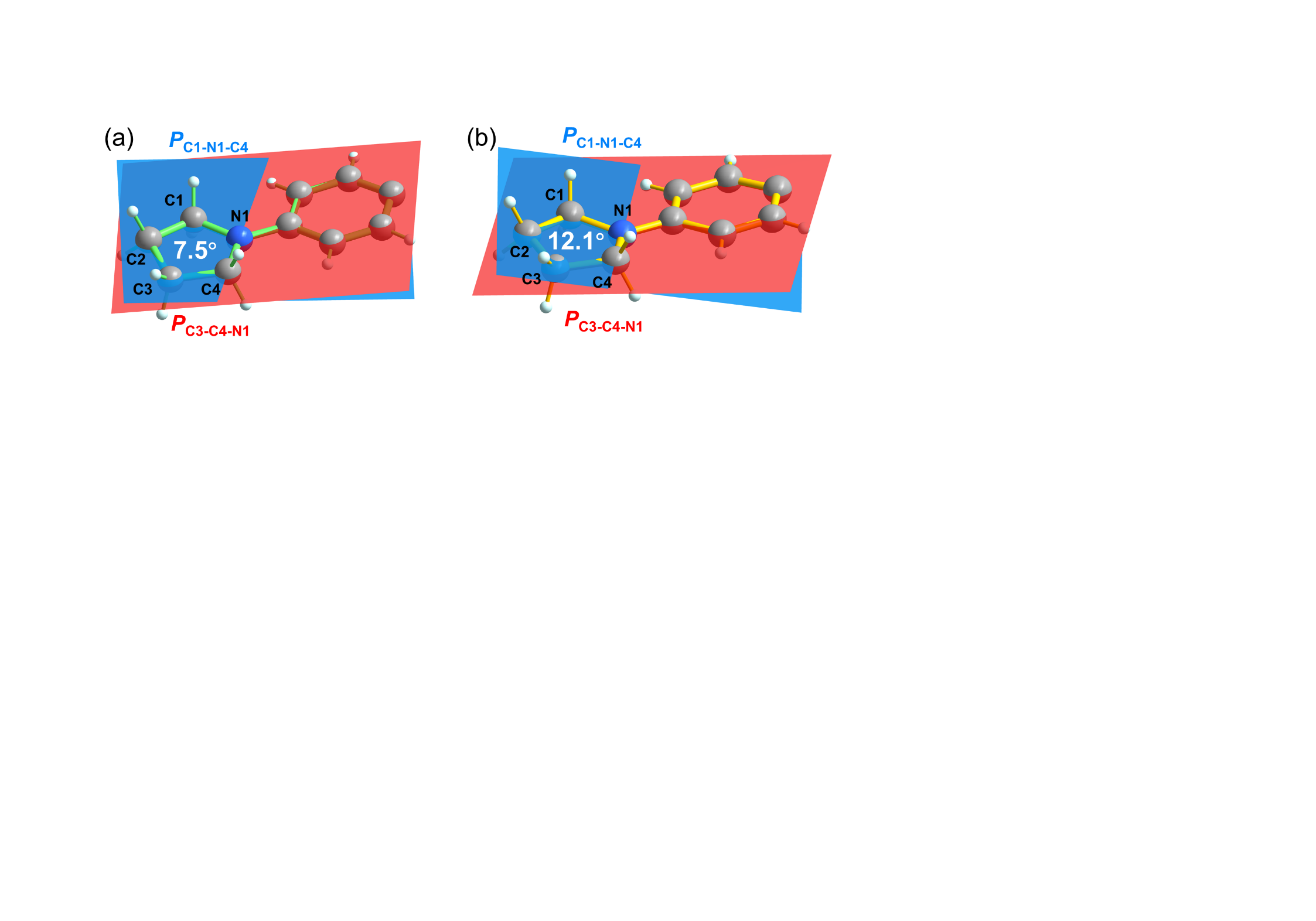


**Figure S4.** The conformations of pyrrolidine ring in **1** at (a) LTP and (b) HTP. Planes *P*_C1-N1-C4_ (blue) and *P*_C3-C4-N1_ (red) represent planes passing through C1-N1-C4 and C3-C4-N1 bonds, respectively.


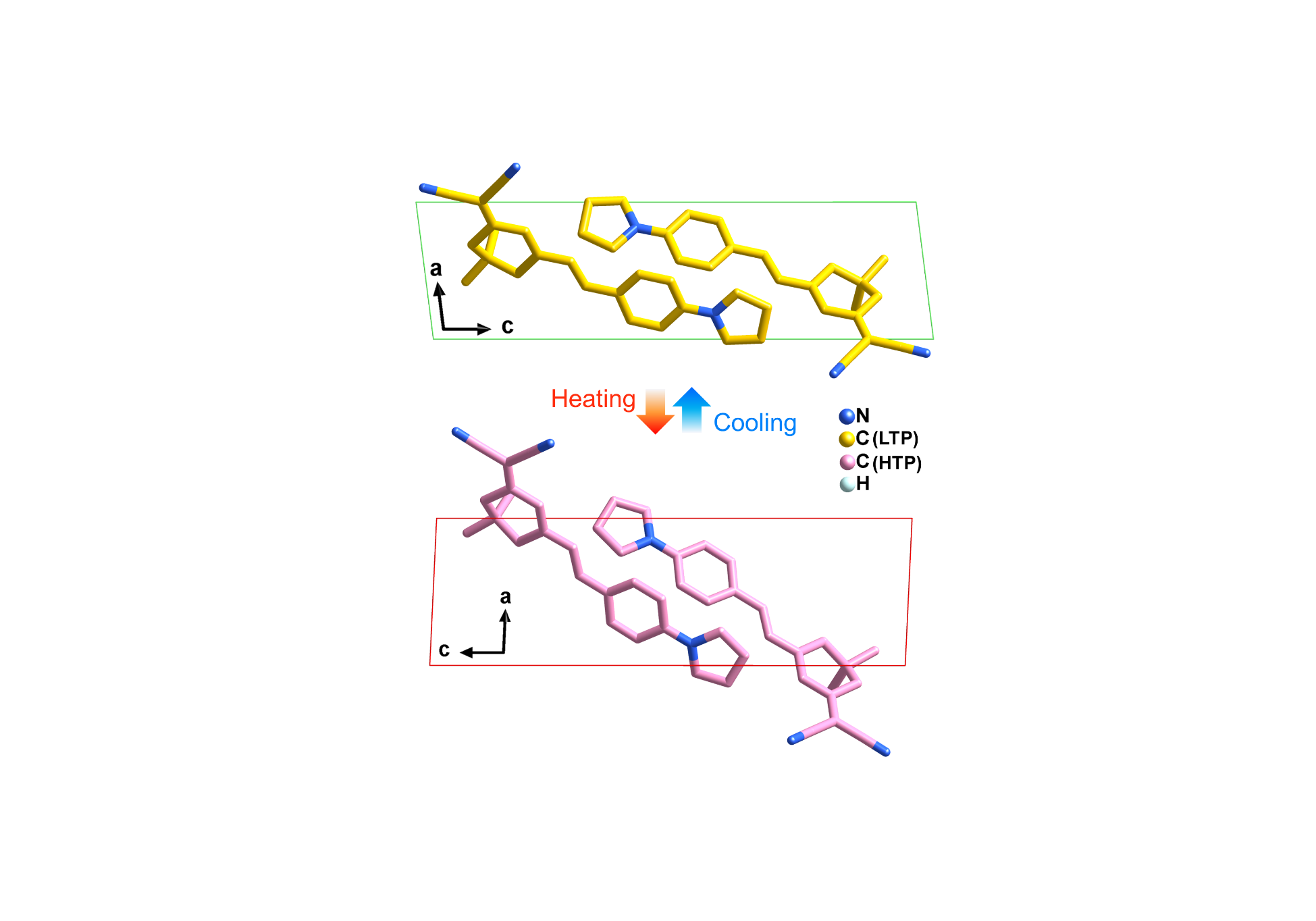


**Figure S5.** Unit cell of **1** at LTP and HTP.


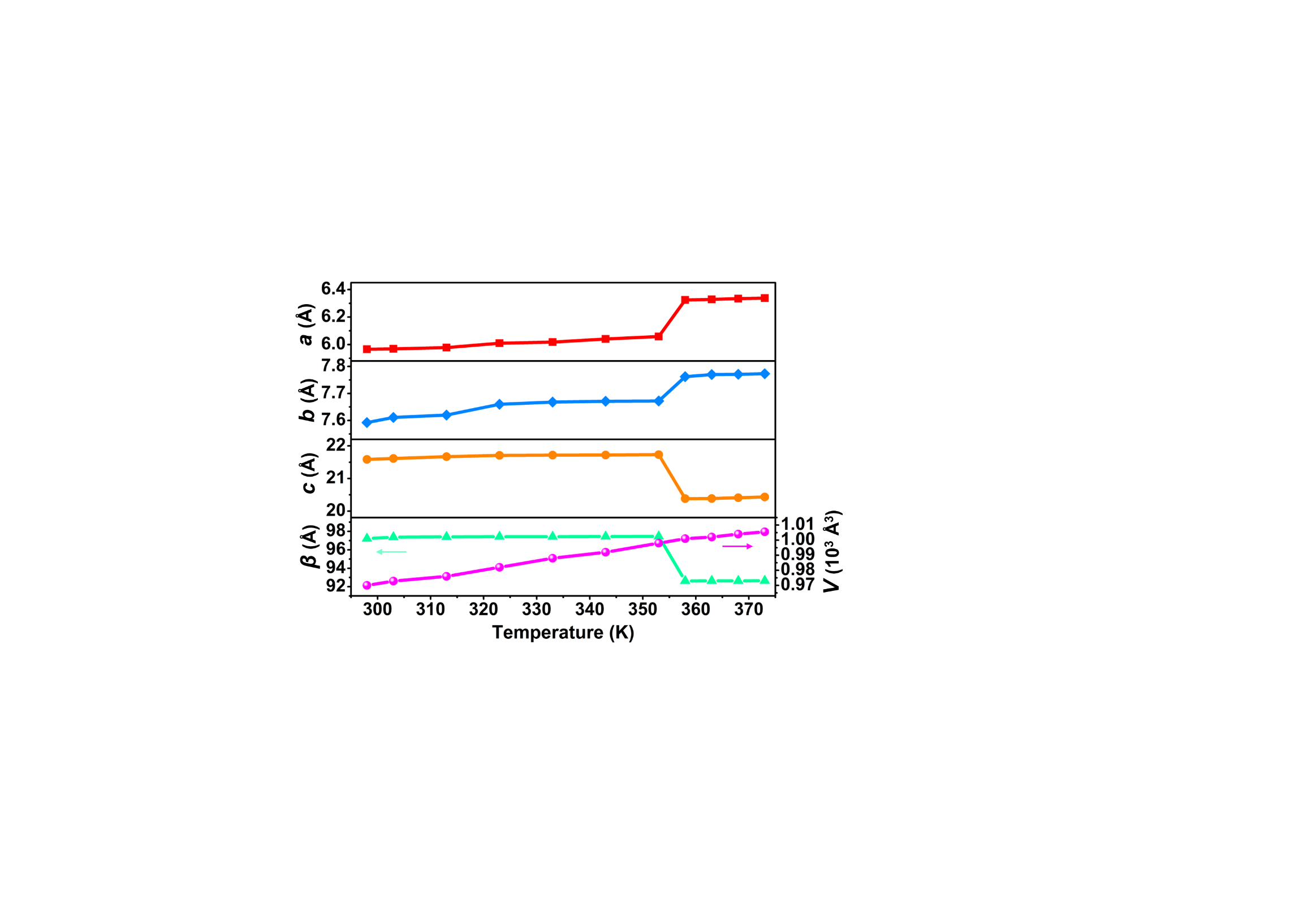


**Figure S6.** Temperature dependence of cell parameters of **1**.


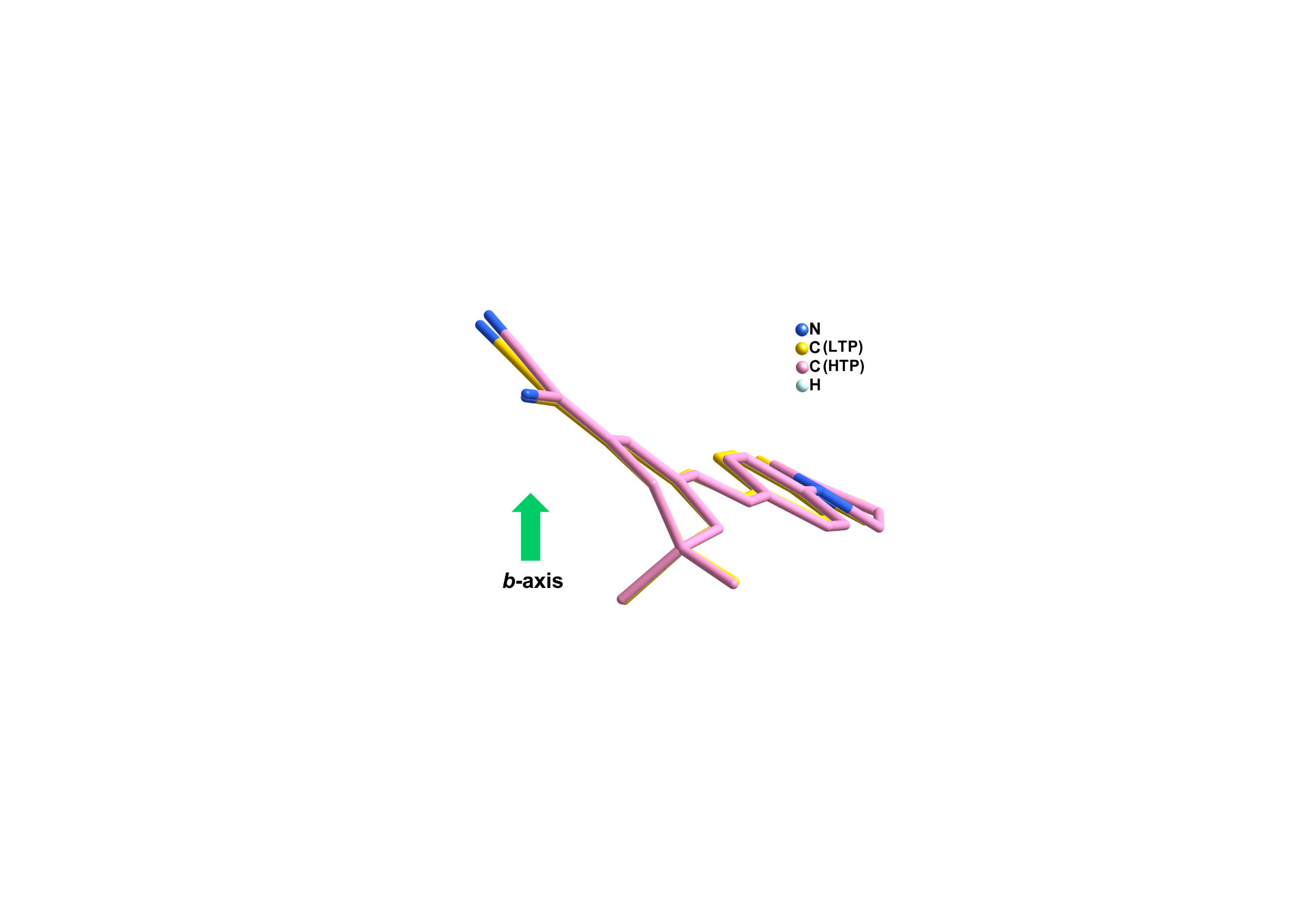


**Figure S7.** The superimposed molecules of **1** to point out the difference in crystalline packing along the *b*-axis at LTP and HTP.


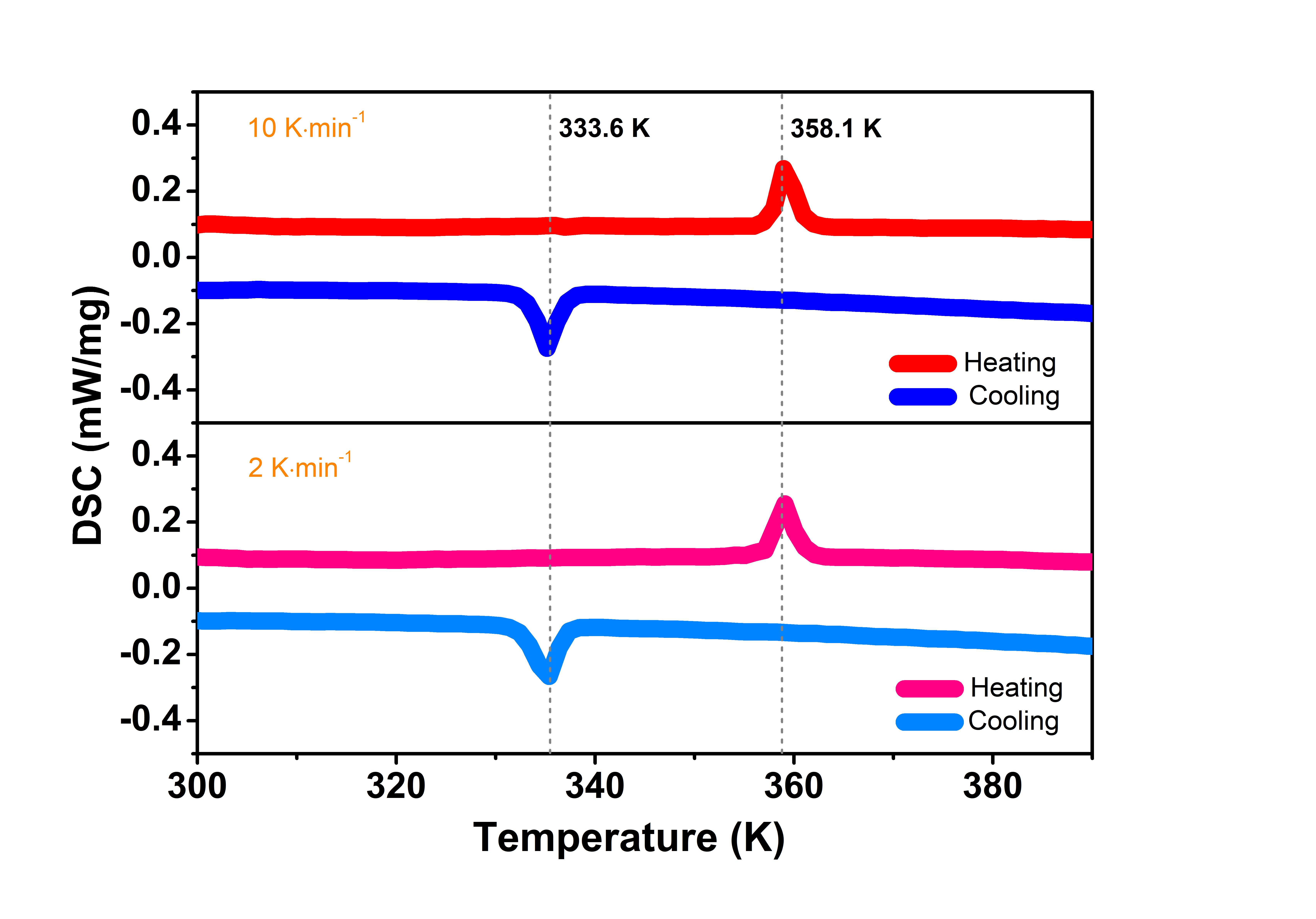


**Figure S8**. DSC curves measured in the heating-cooling cycle at rates of 2 and 10 K∙min^-1^.


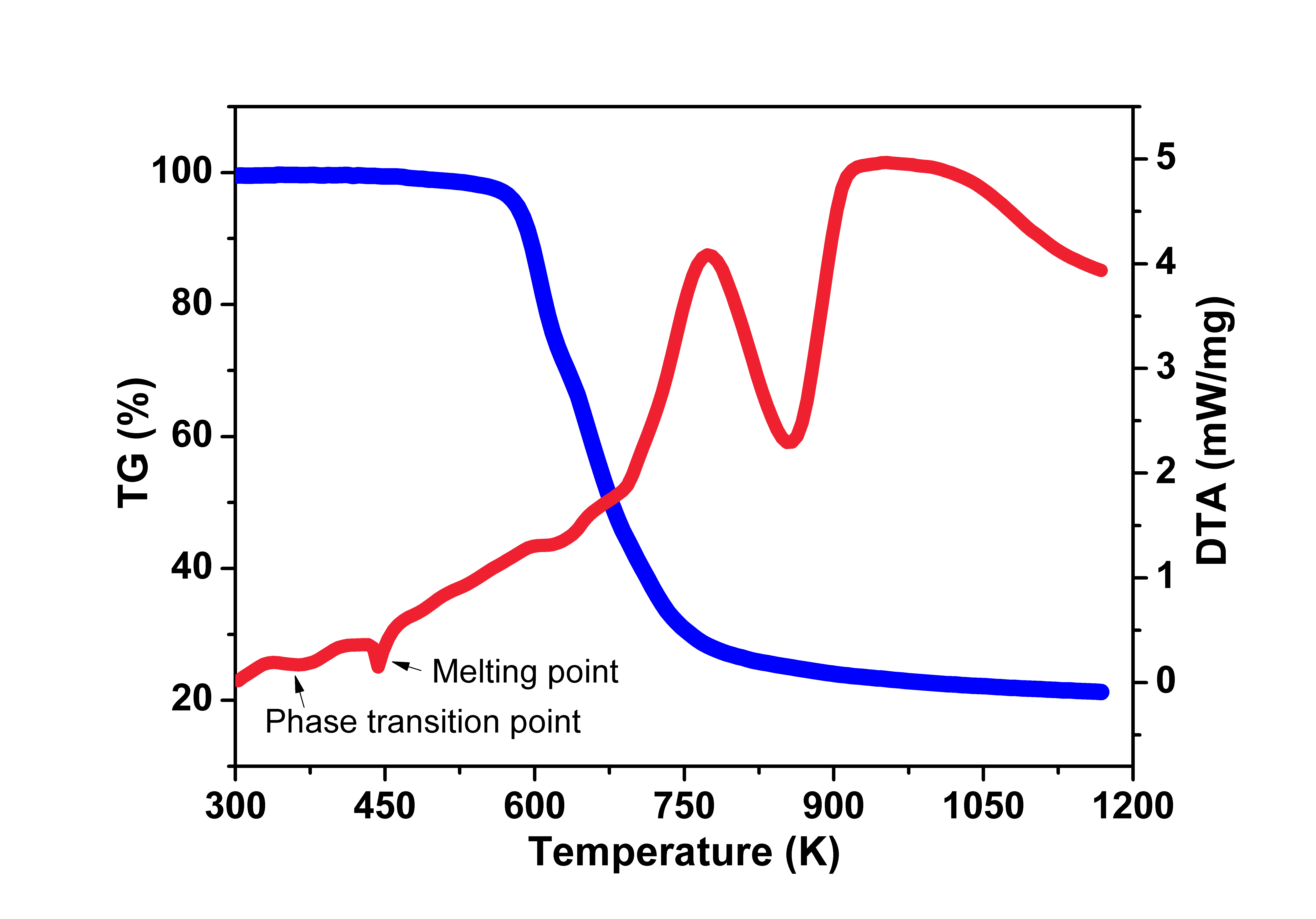


**Figure S9.** TG analysis curve of **1**, indicating a high thermal stability up to ~450 K.


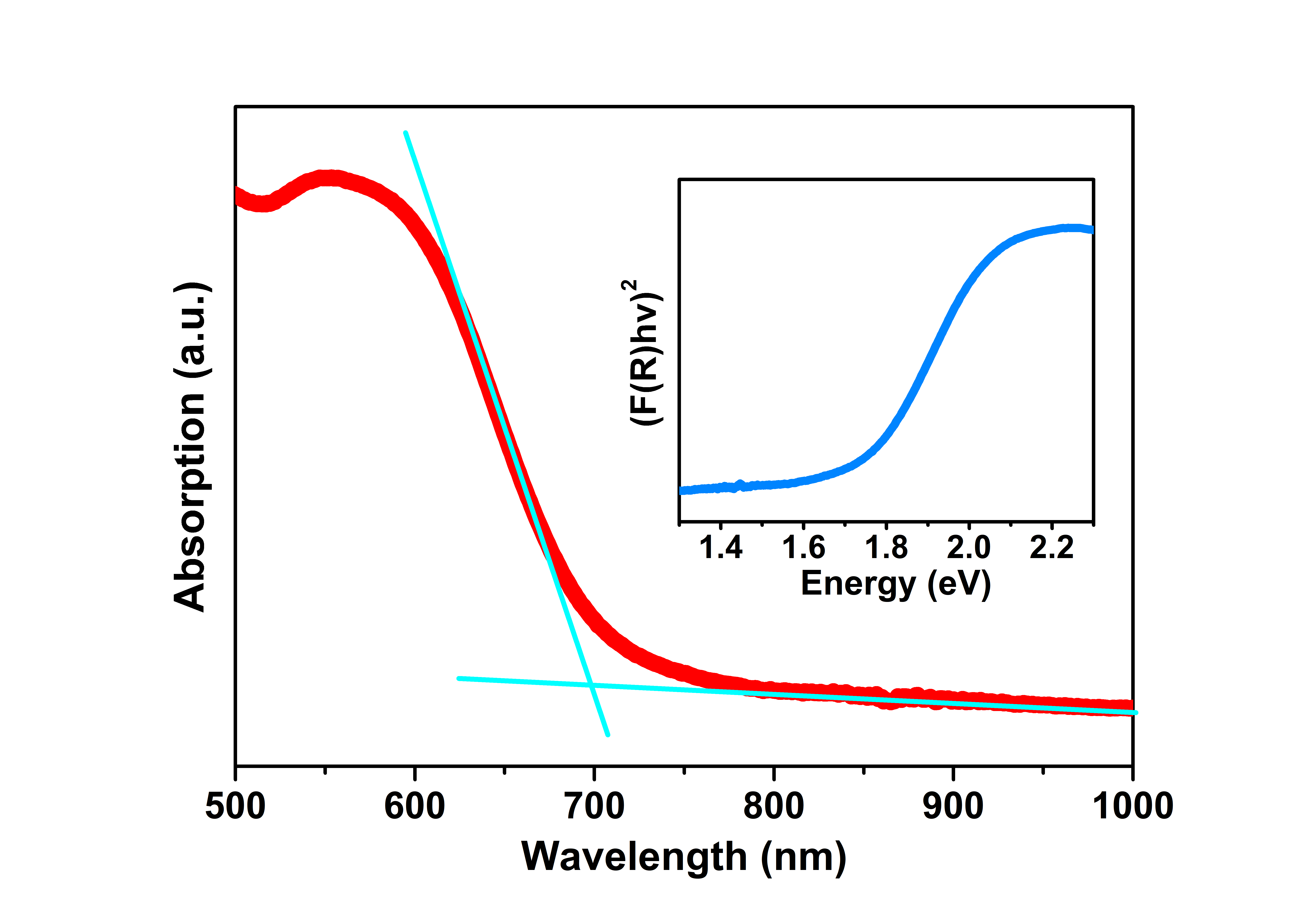


**Figure S10.** Optical absorption spectra of **1**. Inset: calculation of the bandgap.

**Table**

**Table S1.** Crystal data for **1** collected at the low-temperature phase (LTP, 298 K) and high-temperature phase (HTP, 373 K), respectively.

|  | **LTP** | **HTP** |
| --- | --- | --- |
| Empirical formula | C_23_H_25_N_3_ | C_23_H_25_N_3_ |
| Formula weight | 343.46 | 343.46 |
| Temperature/K | 298(2) | 373(2) |
| Crystal system | Monoclinic | Monoclinic |
| Space group | *P*2_1_ | *P*2_1_ |
| *a* (Å) | 5.9659(3) | 6.3378(4) |
| *b* (Å) | 7.5921(3) | 7.7729(6) |
| *c* (Å)  *β* (°) | 21.5863(11)  97.223(5) | 20.4335(13)  92.651(6) |
| *V* (Å^3^) | 969.97(8) | 1005.54(12) |
| D_calca_/Mg·m^-3^ | 1.176 | 1.134 |
| Z | 2 | 2 |
| *µ* (mm^-1^) | 0.537 | 0.518 |
| F(000) | 368.0 | 368.0 |
| 2Θ range /° | 4.126 to 145.46 | 12.184 to 144.352 |
| Index ranges | -7 ≤ *h* ≤ 7, -7 ≤ *k* ≤ 9, -26 ≤ *l* ≤ 25 | -7 ≤ *h* ≤ 7, -9 ≤ *k* ≤ 8, -24 ≤ *l* ≤ 25 |
| Reflections collected | 8459 | 19290 |
| Independent reflections | 2719 [*R*_int_ = 0.0329, *R*_sigma_ = 0.0426] | 3699 [*R*_int_ = 0.0888, *R*_sigma_ = 0.0554] |
| Data/restraints/parameters | 2719/1/238 | 3699/1/237 |
| Goodness-of-fit on *F*^2^ | 1.035 | 0.944 |
| Final R indexes [*I*≥2*σ* (I)] | *R*_1_ = 0.0415, *wR*_2_ = 0.1070 | *R*_1_ = 0.0508, *wR*_2_ = 0.1238 |
| Final R indexes [all data] | *R*_1_ = 0.0582, *wR*_2_ = 0.1183 | *R*_1_ = 0.0830, *wR*_2_ = 0.1416 |

**Table S2.** Bond lengths of crystal **1** at LTP and HTP.

|  |  | **LTP** | **HTP** |
| --- | --- | --- | --- |
| **Atom** | **Atom** | **Length/Å** | **Length/Å** |
| N1 | C5 | 1.363(3) | 1.360(3) |
| N1 | C4 | 1.441(3) | 1.450(4) |
| N1 | C1 | 1.442(4) | 1.449(4) |
| C5 | C6 | 1.412(4) | 1.404(4) |
| C5 | C10 | 1.399(4) | 1.402(4) |
| C8 | C9 | 1.397(4) | 1.387(4) |
| C8 | C11 | 1.459(4) | 1.461(4) |
| C8 | C7 | 1.393(4) | 1.390(4) |
| C12 | C11 | 1.335(4) | 1.326(5) |
| C12 | C13 | 1.447(4) | 1.447(4) |
| C6 | C7 | 1.368(4) | 1.368(4) |
| C9  C14  C14  C4  C22  C22  C13  C18  C15  C15  C23  C23  C2  C2  C16  C19  C17 | C10  C13  C15  C3  N3  C21  C18  C17  C21  C16  C21  N2  C1  C3  C17  C17  C20 | 1.373(4)  1.363(4)  1.421(4)  1.495(4)  1.146(5)  1.431(5)  1.496(4)  1.532(4)  1.373(4)  1.492(4)  1.424(4)  1.142(4)  1.499(5)  1.492(5)  1.530(5)  1.526(4)  1.528(4) | 1.366(4)  1.358(5)  1.418(5)  1.476(5)  1.130(7)  1.440(8)  1.492(5)  1.522(5)  1.375(6)  1.501(6)  1.426(6)  1.139(6)  1.492(5)  1.490(6)  1.516(7)  1.537(6  1.546(6) |

**Table S3.** Bond angles of crystal **1** at LTP and HTP.

|  |  |  | **LTP** | **HTP** |
| --- | --- | --- | --- | --- |
| **Atom** | **Atom** | **Atom** | **Angle/^°^** | **Angle/^°^** |
| C5 | N1 | C4 | 123.0(2) | 122.9(2) |
| C5 | N1 | C1 | 124.2(2) | 124.2(2) |
| C4 | N1 | C1 | 112.7(2) | 111.8(3) |
| N1 | C5 | C6 | 121.6(2) | 121.7(2) |
| N1 | C5 | C10 | 121.9(2) | 122.2(2) |
| C10 | C5 | C6 | 116.5(2) | 116.1(3) |
| C9 | C8 | C11 | 119.7(3) | 119.9(3) |
| C7 | C8 | C9 | 116.0(2) | 115.9(3) |
| C7 | C8 | C11 | 124.4(3) | 124.2(3) |
| C11 | C12 | C13 | 126.3(3) | 126.3(3) |
| C7 | C6 | C5 | 121.4(2) | 121.3(3) |
| C10  C13  N1  N3  C9  C12  C12  C14  C14  C13  C14  C21  C21  N2  C3  N1  C15  C15  C23  C15  C2  C6  C16  C19  C19  C19  C20  C20 | C9  C14  C4  C22  C10  C11  C13  C13  C13  C18  C15  C15  C15  C23  C2  C1  C21  C21  C21  C16  C3  C7  C17  C17  C17  C17  C17  C17 | C8  C15  C3  C21  C5  C8  C18  C12  C18  C17  C16  C14  C16  C21  C1  C2  C22  C23  C22  C17  C4  C8  C18  C18  C16  C20  C18  C16 | 122.6(3)  123.2(3)  105.2(2)  178.5(4)  121.1(3)  126.7(3)  121.0(3)  119.2(3)  119.8(3)  114.3(2)  118.2(3)  120.7(3)  121.1(3)  178.3(4)  106.7(3)  105.5(3)  122.4(3)  122.4(3)  115.2(3)  113.3(2)  107.2(3)  122.3(3)  108.6(3)  110.0(2)  110.1(3)  109.4(3)  109.7(3)  109.1(3) | 122.6(3)  123.7(4)  105.4(3)  179.1(6)  121.5(3)  127.1(3)  120.5(3)  119.2(3)  120.3(3)  113.8(3)  117.1(4)  121.4(4)  121.4(4)  178.4(6)  107.2(3)  105.7(3)  122.4(4)  121.4(5)  116.2(4)  113.5(3)  107.6(3)  122.6(3)  109.4(3)  109.9(3)  110.2(4)  109.2(5)  108.6(3)  109.5(4) |
